# Physiology-Guided Deferral of Percutaneous Coronary Intervention in the Real World

**DOI:** 10.1016/j.jscai.2023.101112

**Published:** 2023-08-05

**Authors:** Ricardo Petraco, Rahul Bahl

**Affiliations:** Department of National Heart and Lung Institute, Imperial College London, London, United Kingdom

**Keywords:** coronary physiology, fractional flow reserve, instantaneous wave-free ratio, percutaneous coronary intervention

It took fractional flow reserve (FFR) nearly 2 decades from its early development in the 1990s to become routinely adopted in clinical practice.^1^ Physicians were previously confident deferring percutaneous coronary intervention (PCI) of intermediate stenoses based on their anatomical severity, and the interventional community only slowly (and somewhat reluctantly) accepted that an invasive, pressure-based physiological index, such as FFR, could trump anatomical guidance. The landmark DEFER study,^2^ however, set the path toward a new era of randomized trials in coronary physiology, making FFR a reference gold standard test for revascularization decision making.

Similar reluctance was observed in 2012 when instantaneous wave-free ratio (iFR) was presented as a nonhyperemic alternative to adenosine-dependent FFR.^3^ Despite demonstrating acceptable numerical agreement, in approximately 20% of cases, iFR and FFR would disagree in stenosis classification and, as such, would give clinicians opposite revascularization guidance as to whether a lesion should be deferred or treated.^4^ The most anxiety-generating mismatch scenario would occur when iFR indicated defer and FFR indicated treat (10% of all cases) as this could mean that a potential future major adverse cardiac event (MACE) was “being missed” by a false-negative nonhyperemic index.

Yet again, data came to the rescue. Both the DEFINE-FLAIR and SWEDEHEART clinical trials demonstrated the safety of PCI guidance with iFR and FFR, with noninferiority between strategies.^5^^,^^6^ Importantly, it became clear that deferring PCI with either iFR or FFR was equally safe and no stenoses were “missed” by resting physiology. Indeed, subsequent studies have since repeatedly demonstrated that iFR deferral is safe in a variety of clinical scenarios, including in the proximal left anterior descending artery.^7^^,^^8^ This noninferiority was maintained in a recent 5-year follow-up of the SWEDEHEART randomized trial^9^; however, although randomized trials offer an ideal platform with which to compare iFR and FFR strategies under blinded and strictly controlled conditions, they suffer from one intrinsic limitation—the inevitable recruitment bias of healthier subjects. Patients in trials do not fully reflect the entire breadth of patients who undergo physiologic assessment in clinical practice, many of whom are older with more comorbidities and likely more severe coronary disease.

In this issue of *JSCAI*, Yndigegn et al^10^ present data from the SWEDEHEART registry, the largest real-world comparison of physiology-guided PCI deferral, with >11,000 patients included. The results agree with those of previous randomized controlled trials and demonstrate equivalence between iFR and FFR when used as tools to defer revascularization. Events rates were similar between groups for MACE (iFR, 26.7%; FFR, 25.9% at 2 years), death (iFR, 10.7%; FFR, 9.6%), and unplanned revascularization (iFR, 17.4%; FFR, 18.1%). This study comes with the known strengths of the SWEDEHEART country-wide registry, which contains an unselected cohort of patients undergoing physiological interrogation of lesions during angiography. No other registry can claim to be more inclusive, with no exclusions from the database possible by researchers. It also has a robust follow-up data collection system, ensuring no loss to follow-up and very little missing data.

One of the most intriguing findings in the study is the high event rates in both groups (26% MACE and 10% deaths within the median 2 years of follow-up). This compares to lower event rates seen in the most recent randomized controlled trials; for example, at a longer follow-up period of 5 years, the iFR-SWEDEHEART trial reported only 20% MACE and 9% deaths. Such differences reflect the differences in inclusion criteria between studies: a truly all-comers population from the SWEDEHEART database vs those selected by investigators to enter a clinical trial. It also demonstrates the importance of real-world data for us to recalibrate expectations of MACE in clinical practice and perhaps tell our patients the real risks of events in the presence of coronary artery disease even in the absence of flow-limiting stenoses.

As an observational registry, the study has limitations too. It is inevitably heterogeneous with respect to types of coronary syndrome, with both stable and acute patients included. Also, it has excluded patients in which both iFR and FFR were measured together, although, unusually, this represented a very small proportion of patients. Finally, the lack of randomization means the operator decided when to use iFR or FFR, introducing biases toward a preferred modality, which are impossible to control for during data analysis.

Although this study adds to the cumulative data demonstrating the safety of PCI deferral based on physiologic guidance, important questions remain only partially answered. First, are all deferred lesions the same? Do the numbers matter? Previous studies have suggested that the lower the FFR on deferred lesions, the higher the risk of MACE,^11^ but to what extent this applies to iFR and to all forms of MACE is unknown. A large registry, such as SWEDEHEART, would be ideal to answer such questions if iFR and FFR were analyzed as continuous variables in their model. Finally, it is important to remember that iFR/FFR measurements only interrogate 1 disease mechanism (ischemia) of 1 domain (epicardial) of the coronary circulation, and events driven by plaque rupture, disease progression, and microcirculatory dysfunction will continue to occur in vessels with high iFR/FFR.

It is now clear that in stable and nonculprit coronary artery disease, tests that discourage the placement of unnecessary stents will likely improve patient prognosis. The SWEDEHEART registry now helps us consolidate the idea that in the real world, across all patients undergoing physiological assessment, PCI deferral can be safely performed with iFR or FFR; however, SWEDEHEART also unequivocally shows us that we must not fall into a false sense of reassurance that all iFR/FFR-negative lesions (and patients) present a low risk of future cardiac events. Optimization of medical therapy and risk factor control, particularly in those patients with left ventricular dysfunction and/or microvascular disease,^12^ must remain the cornerstone of coronary artery disease management.
